# Quality of life in caregivers of aged stroke survivors in southern Brazil: Arandomized clinical trial

**DOI:** 10.1590/1518-8345.5935.3657

**Published:** 2023-01-30

**Authors:** Carla Cristiane Becker Kottwitz Bierhals, Fernanda Laís Fengler Dal Pizzol, Gail Low, Carolina Baltar Day, Naiana Oliveira dos Santos, Lisiane Manganelli Girardi Paskulin

**Affiliations:** 1 Universidade Federal do Rio Grande do Sul, Porto Alegre, RS, Brazil.; 2 University of Alberta, Faculty of Nursing, Edmonton, AB, Canada.; 3 Pontifícia Universidade Católica do Rio Grande do Sul, Escola de Ciências da Saúde e da Vida, Porto Alegre, RS, Brazil.; 4 Grupo Hospitalar Conceição, Hospital Fêmina, Porto Alegre, RS, Brazil.; 5 Universidade Franciscana, Curso de Enfermagem, Santa Maria, RS, Brazil.; 6 Hospital de Clínicas de Porto Alegre, Porto Alegre, RS, Brazil.; 7 Scholarship holder at the Conselho Nacional de Desenvolvimento Científico e Tecnológico (CNPq), Brazil.

**Keywords:** Aged, Caregivers, Clinical Trial, Nursing, Quality of Life, Stroke, Idoso, Cuidadores, Ensaio Clínico, Enfermagem, Qualidade de Vida, Acidente Vascular Cerebral, Anciano, Cuidadores, Ensayo Clínico, Enfermería, Calidad de Vida, Accidente Cerebrovascular

## Abstract

**Objective:**

to evaluate the effect of nursing home care interventions on the quality of life in family caregivers of aged stroke survivors.

**Method:**

a Randomized Clinical Trial, blinded for outcome evaluation. Forty-eighty family caregivers of aged stroke survivors participated in the study. The Intervention Group received three home visits by nurses one month after hospital discharge to provide stroke-related education (i.e., how to access health services and perform care activities) and emotional support. The Control Group received the usual guidance from the health services. Quality of life was assessed using the World Health Organization Quality of Life Assessment (WHOQOL-BREF) instrument and the Old Module(WHOQOL-OLD) 1 week, 2 months, and 1 year after discharge.

**Results:**

the caregivers were mainly women, children, or spouses. The caregivers in the Intervention Group and Control Group did not significantly differ in terms of their Overall Quality of Life at baseline. There was no interaction effect between group allocation and Overall Quality of Life(p=0.625) over time. However, there was an interaction effect for Social Relations(p=0.019) and Autonomy (p=0.004).

**Conclusion:**

the intervention exerted a statistically significant effect on the quality of life of family caregivers with respect to social relationships and autonomy.

**Trial registration:**

NCT02807012.

## Resumo

**Objetivo:**

avaliar o efeito de intervenção educativa domiciliar de enfermagem na qualidade de vida de cuidadores familiares de idosos sobreviventes de acidente vascular cerebral (AVC).

**Método:**

Ensaio Clínico Randomizado, cego para avaliação de resultados. Quarenta e oito cuidadores familiares de idosos sobreviventes de AVC participaram do estudo. O Grupo de Intervenção recebeu três visitas domiciliares de enfermeiros, um mês após a alta hospitalar, para fornecer educação relacionada ao AVC (como acessar os serviços de saúde e realizar atividades de cuidado) e apoio emocional. O Grupo Controle recebeu as orientações habituais dos serviços de saúde. A qualidade de vida foi avaliada usando o instrumento Avaliação da Qualidade de Vida da Organização Mundial da Saúde (WHOQOL-BREF) e o Módulo *Old* (WHOQOL-OLD) em 1 semana, 2 meses e 1 ano após a alta.

**Resultados:**

os cuidadores eram principalmente mulheres, filhos ou cônjuges. Os cuidadores do Grupo Intervenção e do Grupo Controle não diferiram significativamente em termos de Qualidade de Vida Geral no início do estudo. Não houve efeito de interação entre a alocação do grupo e a Qualidade de Vida Geral (p=0,625) ao longo do tempo. No entanto, houve efeito de interação para Relações Sociais (p=0,019) e Autonomia (p=0,004).

**Conclusão:**

a intervenção apresentou efeito estatisticamente significativo na qualidade de vida dos cuidadores familiares no que diz respeito às relações sociais e autonomia.

## Resumen

**Objetivo:**

evaluar el efecto de intervenciones de atención domiciliaria de enfermería sobre la calidad de vida en cuidadores familiares de adultos mayores sobrevivientes de accidentes cerebrovasculares.

**Método:**

Ensayo Clínico Aleatorizado, cegado para la evaluación de los desenlaces. Los participantes del estudio fueron 48cuidadores familiares de adultos mayores sobrevivientes de accidentes cerebrovasculares (ACV). El Grupo Intervención recibió tres visitas domiciliarias a cargo de enfermeros un mes después del alta hospitalaria, en las que se les ofreció instrucción relacionada con ACV (es decir, cómo acceder a los servicios de salud y realizar las actividades inherentes a los cuidados) y apoyo emocional. Al Grupo Control se le brindó la orientación habitual de los servicios de salud. La calidad de vida se evaluó mediante el instrumento *World Health Organization Quality of Life Assessment* (WHOQOL-BREF) y el módulo *Old*(WHOQOL-OLD) 1semana, 2meses y 1año después del alta.

**Resultados:**

en su mayoría, los cuidadores fueron mujeres, hijos o cónyuges. Los cuidadores de los grupos Intervención y Control no presentaron diferencias significativas en términos de su Calidad de Vida general de base. La intervención no ejerció ningún efecto entre la asignación a los grupos y la Calidad de Vida general(p=0,625) con el transcurso del tiempo. Sin embargo, la intervención sí tuvo efecto sobre las Relaciones Sociales (p=0,019) y la Autonomía(p=0,004).

**Conclusión:**

la intervención ejerció un efecto estadísticamente significativo sobre la calidad de vida de los cuidadores familiares con respecto a las relaciones sociales y a la autonomía.

**Highlights**

(1) Presents effect related to the caregivers’ quality of life (social and autonomy).

(2) Educational interventions should be focused on post-discharge care activities.

(3) Educational interventions should be focused on the emotional of family caregivers.

(4) The findings provide recommendations for nurses and policymakers.

## Introduction

Stroke is one of the main causes of death worldwide and the most prevalent cerebrovascular disease among aged people^1^. Stroke survivors often experience significant physical and cognitive sequelae that can hinder activities of daily living^2^. Consequently, these individuals need support from other people. Family caregivers (including family members, friends, and neighbors) tend to provide the bulk of care, ranging from household chores to personal care, such as hygiene, medication and feeding^3^ ^-^ ^4^.

Many family caregivers feel unprepared to care for stroke survivors, as they rarely receive sufficient training from health professionals^5^. Consequently, family caregivers experience poorer Quality of Life (QoL)^2^ ^,^ ^6^ ^-^ ^7^. Support and education for family caregivers should be part-and-parcel of routine Nursing care^8^, particularly with respect to practical education aimed at improving their skills. In this sense, educating family members on the daily caregiving tasks for stroke patients can improve QoL in family caregivers^9^. Nurses play a fundamental role in educating stroke survivors and their family caregivers throughout hospitalization, as well as in preparation for and after discharge.

QoL is defined as “the individuals’ perception of their position in life in the context of the culture and value systems in which they live and in relation to their goals, expectations, standards, and concerns”^10^. Lack of training can exert a negative impact on a family caregiver’s QoL. A Randomized Controlled Trial (RCT) conducted in England with 300stroke patients and their caregivers found remarkable disparities in QoL among trained versus untrained caregivers (EuroQol score 80 *vs*. 70; p=0.001)^11^. Some systematic reviews of interventions for family caregivers and stroke survivors indicate that educational programs can improve their QoL related to psychological health, prevent problems due to burden and reduce the caregivers’ depression and burden levels^12^ ^-^ ^14^. In addition, it highlights the importance of developing interventions at the caregivers’ homes, for being the environment where they have more problems and lack of support^13^.

Researchers in China, England, United States of America, Germany, and Hong Kong have long argued for the merits of, and thus have developed, programs guiding and preparing caregivers, with a view to improving their QoL^3^ ^,^ ^11^ ^,^ ^15^ ^-^ ^17^. Accordingly, essential elements have included transitional care programs, hospital discharge planning, telephone calls, home visits(HVs) and multidimensional skills development, psychoeducation, and peer support.

Educational interventions to support health professionals and managers regarding home care practices are common outside Brazil. Home care services are not yet fully consolidated in the Brazilian health policy and informal caregiving has not yet entered the Brazilian public policy radar^18^. To the present day, there are no studies assessing the effectiveness of Nursing interventions on Quality of Life in caregivers of aged stroke survivors. According to Hinrichs-Krapels and Grant^19^, effectiveness in research considers whether a proposed intervention produces the expected outcome and/or societal benefits or impact.

The existing studies are descriptive or verify the association of QoL with the caregivers’ sociodemographic characteristics^20^ ^-^ ^21^. Therefore, there is lack of randomized clinical trials that test whether the care educational practices performed by nurses affect the QoL of these caregivers. As such, intervention studies are needed to evaluate the effectiveness of Nursing care educational practices for family caregivers and to strengthen the provision of home healthcare. We hypothesized that family caregivers of aged stroke survivors receiving an educational Nursing intervention would report higher QoL levels than those receiving usual follow-up care. The objective of this study is to evaluate the effect of a Nursing Home Care Intervention on the QoL of family caregivers of aged stroke survivors.

## Method

### Design

An RCT, blinded for outcome evaluation. This study is part of a larger RCT called “Nursing Home Care Intervention Post Stroke” (SHARE), registered in ClinicalTrials.gov(NCT02807012). The protocol of this study was methodologically performed and previously published to ensure replicability^22^. This research presents QoL as the primary outcome. Additionally, this RCT has another primary outcome: the caregivers’ burden^23^. The secondary outcomes are as follows: use of health services and rehospitalization^24^, as well as functional capacity of stroke survivors^23^.

### Setting

The study participants were family caregivers of aged stroke survivors from the Stroke Special Care Unit (SCU-Stroke) of *Hospital de Clínicas de Porto Alegre* (HCPA). The educational intervention was performed in the participants’ homes one month after discharge.

Porto Alegre is the capital city of the state of Rio Grande do Sul (Brazil) and is considered the second Brazilian capital with the highest number of older adults, representing 14.05% of the population^25^. HCPA is one of the reference hospitals in caring for stroke patients. SCU-Stroke was created in 2013 and consists in a multidisciplinary team, including physicians, nurses, pharmacists, nutritionists, physiotherapists, speech therapists, social workers, and psychologists.

### Population, eligibility criteria and sampling

This study was conducted with family caregivers of stroke survivors aged 60 years old and over. The stroke survivors included in the study were those with a minimum score of 2 (no significant disability despite the symptoms; able to carry out all usual duties and activities) in The Modified Rankin Scale (mRankin)^26^ and a maximum score of 5 (severe disability; bedridden, incontinent, and requiring constant Nursing care and attention) at hospital discharge. The mRankin scale determines how disabled or dependent a stroke survivor is in their daily activities. The additional criterion was the following: the stroke survivor’s house is located within 20 km of the SCU. The eligibility criteria for caregivers were as follows: (a) 18+ years of age; (b) non-kin and kin family members; (c) unpaid caregivers; and (d) declaring themselves responsible for the bulk of care. Stroke survivors with planned admissions to a Nursing home or Home Care Service (HCS) were excluded, so too were caregivers refusing HVs from the research team.

Sample size in this RCT was estimated based on an RCT showing 10-point improvements in caregivers’ QoL^11^. Based on an *a priori* 95% confidence level, a statistical power of 80%, a minimum effect size of 0.8 QoL standard deviations between groups, and 20% oversampling (for a possible attrition rate), it was necessary to recruit at least 48 family caregivers. Among the 471 patients admitted to SCU-Stroke during the recruitment period, 245 were eligible to take part in the RCT. Among all eligible participants, 197 did not meet our inclusion criteria. Thus, our final study sample (n=48) was randomly allocated to the Intervention Group(IG) (n=24) and to the Control Group (CG) (n=24).

### Randomization and blinding

Randomization was performed using a list generated by the *randomisation.com* website, which was arranged in a numbered order with each number assigned at random, either to the IG or to the CG. After collecting baseline data, research assistants (undergraduate students) contacted a nurse who did not participate in the intervention and was responsible for the generated list. Subsequently, this nurse allocated the participants to the intervention and informed the interventionist nurses (INs). As soon as the participants were accepted into the study, they were randomly allocated. Only the participants assigned to the IG were known to the INs. The research assistants were blinded to the participants’ allocation group for evaluation at baseline, as well as 2 months and 1 year after discharge. Risk of bias is related to non-blinding of the outcome evaluators. Therefore, the INs made telephone calls to all caregivers 2 days before the outcome assessment to reinforce them not to mention whether or not they received the intervention.

### Control Group

During hospitalization and at discharge, the family caregivers received usual care from a multidisciplinary team in SCU-Stroke. Additionally, they underwent follow-up from their respective health service networks, which typically includes general information about the disease and some aspects inherent to care, such as drug administration and nutrition^22^.

### Intervention

The IG received usual care and the SHARE intervention, which included three HVs from two trained nurses approximately 14, 21 and 30 days after discharge. The INs engaged in a dialogic process with the family caregivers which, in turn, stimulated reflective thinking and shared answers^27^. This better understanding of the everyday life demands and resources available in the caregivers’ homes allowed the INs to more aptly guide the family caregivers to the caregiver role^22^. For example, the caregivers were asked about their feelings, doubts and resources (diet support, hygiene supplies, type of bed, access to a walker, etc.) so that they could be instructed accordingly. In essence, the INs were able to tailor their explanations of how survivor care could be best delivered in the caregivers’ homes.

Caregiver education was provided in observing a recommendation that includes, for example, how to safely prepare food, and adaptive clothing^28^. The caregivers’ educational needs were also selected based on the stroke survivors’ baseline Functional Independence Measure (FIM) scores. All such scores could range from 1 (total dependence) to 6 (modified independence).

### Study variables and instruments

The stroke survivor data collected prior to discharge pertained to identification (name, address and contact details), sociodemographic data (age, biological sex, schooling, marital status, family income and professional status) and physical health (type of stroke, comorbidities, mRankin and the FIM scores). The caregiver data included sociodemographic characteristics (age, biological sex, schooling and marital status), health status (health problems and morbidities) and caregiver status (relationship and living arrangements with the stroke survivor, days spent caring for the stroke survivor, previous caregiving experience and type of help received from others). The primary outcome of family caregiver’s QoL was assessed using the World Health Organization Quality of Life-Bref (WHOQOL-BREF) instrument and the WHOQOL-OLD module for caregivers aged at least 60 years old. The stroke survivors’ data (identification, sociodemographic data and physical health) and the caregivers’ data (sociodemographic characteristics, health and caregiver status) were collected using a specific questionnaire prepared for this study.

Functional capacity of the stroke survivors was assessed by means of FIM. This is a measure of how physically independent aged stroke survivors are^29^. There are six dimensions pertaining to self-care, sphincter control, transfer, locomotion, communication and social cognition. The dimensions’ scores can range from 1 (total dependence) to 7 (total independence). The overall FIM scores can range from 18 to 126. Lower scores indicate higher physical dependence. In this RCT, internal consistency reliability of FIM was α=0.775 at baseline, α = 0.829 at month 2, and α = 0.838 at year 1.

The 24-item WHOQOL-BREF instrument captures QoL across four domains: physical, psychological, social relationships, and environment^30^. The respondents also rate their Overall QoL and General Health. The scores are derived by adding scores for each 5-point Likert scale item germane to each parent domain. As such, the higher the domain score, the better the QoL^31^. Caregivers aged 60 and older also answered WHOQOL-OLD. This adjunct module concerns sensory abilities, autonomy, past, present and future activities, social participation, death and dying, and intimacy. Higher WHOQOL-OLD scores represent higher QoL^32^.

### Data collection

The data were collected between May 2016 and July 2018. All caregivers were visited at their homes by two research assistants, the same who collected baseline caregiver QoL data using WHOQOL-BREF and adjunct WHOQOL-OLD. The stroke survivors were also assessed using FIM. Afterwards, a nurse, who was a research team member but not an IN, randomly allocated 24 caregivers to the IG and another 24 to the CG.

The participants in the IG received three additional HVs, one week apart. All IG and CG participants received HVs from research assistants to collect caregiver QoL data two months (month 2) and one year (year 1) after discharge. All such data were collected from allocation-blinded research assistants.

### Statistical analysis

The analyses were performed with intention to treat (ITT). Regardless of the treatment (if any) they received, all randomized participants were included in the statistical analysis and examined according to the group to which they were originally allocated^33^. Missing data were imputed by the LOCF (Last-Observation-Carried-Forward) method^34^ ^-^ ^35^.

The analyses were conducted using the Statistical Package for the Social Sciences(SPSS), version 21.0. Depending on the measurement level, the Student’s t, Mann-Whitney’s U, Pearson’s Chi-square or Fisher’s Exact tests were used to generate and compare family caregivers’ and stroke survivors’ characteristics at baseline. A Generalized Estimating Equations (GEE) model was then employed to capture the effects of the SHARE intervention on the caregivers’ WHOQOL-BREF and WHOQOL-OLD scores over time. “Over time” comparisons were made 7 days after discharge (baseline) versus 2 months after discharge, and at baseline versus 1 year after discharge. We controlled for remarkably different (p<.15) CG and IG family caregiver characteristics, with these possibly including survivor’s marital status, caregiver-survivor relationships, time living with the survivor, and days spent as a family caregiver.

### Validity and reliability

The Brazilian version of WHOQOL-BREF presented good performance concerning internal consistency (α = 0.91), discriminant validity, criterion validity, concurrent validity and test-retest reliability (correlational coefficient scores above 0.7)^30^. The Brazilian WHOQOL-OLD module presented good internal consistency (α = 0.885), concurrent validity and test-retest reliability (overall Pearson’s correlation coefficient = 0.820)^32^. 

The Cronbach’s α coefficients of WHOQOL-BREF in this study at baseline were as follows: Physical Health (α = 0.833), Psychological (α = 0.666), Social Relationships (α = 0.507), Environment (α=0.716), and Overall QoL (α = 0.847). At month 2, the internal consistency reliability coefficients (Cronbach’s α) were as follows: 0.776, 0.598, 0.750, 0.651 and 0.803, respectively. At year 1, these coefficients (Cronbach’s α) were 0.831, 0.809, 0.604, 0.706 and 0.909.

Regarding WHOQOL-OLD, the Cronbach’s α coefficients at baseline were the following: Sensory Abilities (α = 0.214), Autonomy (α = 0.558), Past, Present and Future Activities (α = 0.695), Social Participation (α = 0.497), Death and Dying (α = 0.827), Intimacy (α = 0.936) and Overall (α = 0.785). At month 2, they were 0.410, 0.521, 0.389, 0.414, 0.841, 0.832 and 0.681, respectively. At year 1, these coefficients were 0.583, 0.589, 0.475, 0.673, 0.665, 0.803 and 0.763. 

### Fidelity of the study

The protocol of this study was documented to guarantee reproducibility^22^. All members of the research team (researchers, assistants, interventionist nurses, and a nurse not involved in the intervention) were well-instructed as to the study protocol. The same research assistants collected QoL data from the same participants (before and after the intervention). Comprehensive expectations and objectives of the study were clearly described and explicitly documented for all research team members and participants.

An application guide, containing instructions on how to administer and score WHOQOL and FIM, was made available to the research assistants. All INs had access to the care protocol developed^28^ from the literature and in consensus with an expert committee.

The HVs were scheduled with the caregivers by phone according to their availability. To mitigate errors in study data entry, the research assistants independently entered the survey answers into an Excel spreadsheet, with cross-checking for inconsistencies by an IN.

### Ethical considerations

The participants signed an Informed Consent Form and with assurance of voluntary participation and anonymity. They would be able to withdraw from the study without prejudice, including access to any public health services. No physical harms were anticipated. The study was approved by the institution’s Research Ethics Committee (#16-0181).

## Results

**Figure 1:**
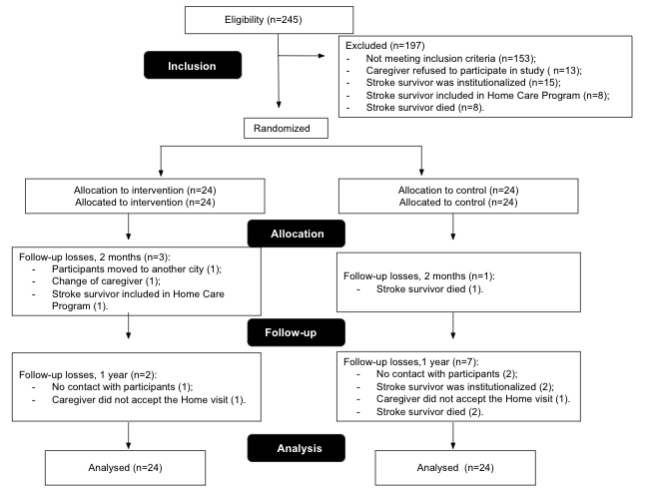
RCT Diagram According to the Consolidated Standards of Reporting Trials(CONSORT)

The RCT diagram according to the Consolidated Standards of Reporting Trials (CONSORT) is shown in Figure 1. 

The stroke survivors’ sociodemographic characteristics and health conditions are shown in Table 1. The survivor groups differed based on marital status (*p*=.021) alone. The family members’ sociodemographic characteristics and health and caregiving status in the IG and CG were similar (Table 2).

**Table 1:** Sociodemographic characteristics and health status of the stroke survivors (n=48). PortoAlegre, RS, Brazil, 2018

Variables	CG* (n=24)	IG^†^ (n=24)	*p*
Age (years old)^‡^	74.3± 8.5	73.0 ± 10.3	0.659
**Biological sex (%)^§^**			
Female	12 (50.0)	14 (58.3)	0.772
Marital status (%)^§^			0.021
Married/With a partner	16 (67)	7 (29)	
Single	8 (33)	17 (71)	
Schooling (years)^||^	5 (3-8)	4 (2.3-5.0)	0.156
Professional status (%)^§^			0.165
Active	3 (12.5)	2 (8.3)	
Not active	21 (87.5)	22 (91.7)	
Family income^||^ (minimum wage)^¶^	1,760 (1,760-2,200)	2,100 (1,715-3,410)	0.302
Stroke (%)^§^			1.000
Ischemic	23 (95.8)	23 (95.8)	
**Morbidity (%)^§^**			
Arterial hypertension	22 (91.7)	20 (83.3)	0.666
Cardiac diseases	11 (45.8)	11 (45.8)	1.000
Vascular problems	4 (16.6)	4 (16.6)	1.000
mRankin score at discharge (%)^§^			0.980
Slight disability	3 (12.5)	3 (12.5)	
Moderate disability	5 (20.8)	6 (25)	
Moderately severe disability	10 (41.7)	10 (41.7)	
Severe disability	6 (25)	5 (20.8)	
**Total FIM functional capacity^‡^**			
Baseline	60.0±22.2	60.6±16.9	0.895
2 months	78.7±25.8	76.6±22.7	0.779
1 year	86.6±22.2	81.2±24	0.501

*CG = Control Group; ^†^IG = Intervention Group; ^‡^Mean ± standard deviation, independent ttest; ^§^Absolute number and (%), Pearson’s Chi-square test and Fisher’s Exact test; ^||^Median and 25^th^ and 75^th^ percentiles, Mann-Whitney test; ^¶^Minimum wage at data collection was R$880.00 in 2016/Brazil

**Table 2:** Characteristics of the family caregivers (n=48). Porto Alegre, RS, Brazil, 2018

Variables	CG* (n=24)	IG^†^ (n=24)	*p*
Age (years old)^‡^	53.54±14.05	53.38±11.91	0.965
**Biological sex (%)^§^**			
Female	19 (79.2)	23 (95.8)	0.188
Schooling (years)^||^	8 (4-11)	10 (5-11)	0.249
Professional status (%)^§^			0.555
Active	8 (33.3)	11 (45.8)	
Not active	16 (66.7)	13 (54.2)	
Marital status (%)^§^			0.182
Married/With a partner	20 (83.3)	16 (66.7)	
Single	4 (16.7)	8 (33.4)	
Relationship to the older adult (%)^§^			0.106
Child	6 (25)	13 (54.2)	
Spouse	13 (54.2)	7 (29.2)	
Other	5 (20.8)	4 (16.7)	
Health problem (yes)^§^	17 (70.8)	19 (79.2)	0.739
**Morbidity (%)^§^**			
Arterial hypertension	10 (41.7)	10 (41.7)	1.000
Diabetes	5 (20.8)	3 (12.5)	0.701
Musculoskeletal disease	2 (8.3)	4 (16.7)	1.000
Living with the older adult (yes)^§^	22 (91.7)	20 (83)	0.523
Time living with older adult (years)^||^	12.5 (3.1-31.5)	17.2 (0.01-34.5)	0.110
Days as caregiver^||^	20.5 (13.25-103)	14.5 (5-43)	0.117
Previous experience in care (yes)^§^	17 (70.8)	14 (58.3)	0.547
Received help for care after discharge (yes)^§^	22 (95.7)	19 (90.5)	0.599
**Type of help (%)^§^**			
Instrumental	22 (95.7)	19 (90.5)	0.599
Emotional	17 (73.9)	12 (57.1)	0.241
Financial	9 (39.1)	11 (52.4)	0.378

*CG = Control Group; ^†^IG = Intervention Group; ^‡^Mean ± standard deviation, independent ttest; ^§^Absolute number and (%), Pearson’s Chi-square test and Fisher’s exact test; ^||^Median and 25^th^ and 75^th^ percentiles, Mann-Whitney test

The effects of the SHARE intervention on the WHOQOL-BREF scores are presented in Table 3. Overall QoL was similar between IG and CG caregivers along time. Between month 2 and year 1, the overall QoL scores were statistically significantly lower (*p*=.018) among the CG caregivers. 

There were statistically significant changes in the Social Relationships scores over time. The CG caregivers had a significantly lower QoL between baseline and year 1 (*p*=.002) and between month 2 and year 1 (*p*<.001). The IG caregivers presented much higher QoL levels between month 2 and year 1 (*p*=.019).

The environmental QoL scores among the CG caregivers were generally higher at baseline. At month 2, there was a statistically significant (*p*=.037) drop in the QoL of CG caregivers. Hence, the absence of a robust group QoL interaction over time.

Baseline analyses of WHOQOL-OLD were undertaken using data from nine CG and six IG caregivers. It is important to mention that, during the intervention, the study had losses of participants: at month 2, one CG and two IG caregivers were no longer able to participate and, at year 1, one IG caregiver withdrew from the study. However, using the LOCF method and ITT, all caregivers from the CG (n=9) and the IG (n=6) were analyzed, regardless of the losses.

**Table 3:** Effects of SHARE on the caregivers’ WHOQOL-BREF scores (n=48). Porto Alegre, RS, Brazil, 2018

Domain*	Item	CG^†^ (n=24)	IG^‡^ (n=24)	*p* ^§^
Physical health	Baseline^||^	61.6±3.5	63.9±4.0	0.676
	Month 2^||^	63.3±3.6	57.8±3.4	0.295
	Year 1^||^	57.1±3.7	58.7±3.1	0.738
	Month 2 - Baseline	1.73 (-7.01 to 10.5)	-6.17 (-13.6 to 1.29)	0.267
	*p* ^¶^	0.698	0.105	
	Year 1 - Month 2	-6.22 (-14.0 to 1.57)	0.99 (-5.46 to 7.44)	
	*p* ^¶^	0.118	0.763	
	Year 1 - Baseline	-4.49 (-12.6 to 3.59)	-5.17 (-13.5 to 3.16)	
	*p* ^¶^	0.276	0.224	
Psychological	Baseline^||^	65.6±3.0	63.5±3.0	0.656
	Month 2^||^	63.9±2.6	60.4±2.2	0.370
	Year 1^||^	61.5±2.7	62.7±3.5	0.803
	Month 2 - Baseline	-1.68 (-6.95 to 3.57)	-3.10 (-9.44 to 3.24)	
	*p* ^¶^	0.530	0.338	
	Year 1 - Month 2	-2.35 (-6.93 to 2.22)	2.22 (-5.15 to 9.60)	0.563
	*p* ^¶^	0.314	0.555	
	Year 1 - Baseline	-4.04 (-10.1 to 2.06)	-0.88 (-10.1 to 8.31)	
	*p* ^¶^	0.195	0.851	
Social relationships	Baseline^||^	69.2±3.1	63.4±3.1	0.197
	Month 2^||^	66.7±2.8	60.1±3.6	0.162
	Year 1^||^	56.7±3.2	62.4±2.2	0.151
	Month 2 - Baseline	-2.57 (-9.88 to 4.73)	-3.39 (-11.7 to 4.92)	
	*p* ^¶^	0.490	0.424	
	Year 1 - Month 2	-9.93 (-14.7 to -5.18)	2.31 (-5.57 to 10.2)	0.019
	*p* ^¶^	<0.001	0.565	
	Month 2 - Baseline	-12.5 (-20.4 to -4.57)	-1.07 (-8.38 to 6.24)	
	*p* ^¶^	0.002	0.773	
Environment	Baseline^||^	56.3±3.2	50.4±2.4	0.161
	Month 2^||^	59.5±2.2	52.7±2.4	0.037
	Year 1^||^	60.8 ±2.2	54.6± 2.7	0.072
	Month 2 - Baseline	3.17 (-1.49 to 7.84)	2.22 (-3.78 to 8.24)	
	*p* ^¶^	0.182	0.464	
	Year 1 - Month 2	1.37 (-2.18 to 4.93)	1.91 (-2.85 to 6.67)	0.961
	*p* ^¶^	0.450	0.432	
	Year 1 - Baseline	4.54 (-1.15 to 10.2)	4.14 (-2.61 to 10.8)	
	*p* ^¶^	0.117	0.229	
Overall	Baseline^||^	63.2±4.2	59.6±3.1	0.530
	Month 2^||^	68.4±3.8	60.3±3.3	0.125
	Year 1^||^	59.3±3.3	57.5±4.6	0.752
	Month 2 - Baseline	5.25 (-3.58 to 14.1)	0.67 (-6.90 to 8.24)	
	*p* ^¶^	0.244	0.862	
	Year 1 - Month 2	-9.3 (-16.7 to -1.6)	-2.77 (-14.6 to 9.02)	0.625
	*p* ^¶^	0.018	0.644	
	Year 1 - Baseline	-3.88 (-13.4 to 5.64)	-2.11 (-11.1 to 6.9)	
	*p* ^¶^	0.424	0.646	

*Estimated means ± standard error; ^†^CG = Control Group; ^‡^IG = Intervention Group; ^§^Effect of the intervention between the groups by the General Estimated Equations(GEE) model with Least Significant Difference (LSD) adjustment. Adjusted p for the following variables: marital status of the aged, relationship with the aged, time living with the aged and days as caregiver; ^||^Baseline: 7 days after discharge; Month 2: two months after discharge; Year 1: one year after discharge; ^¶^Effect of the intragroup GEE intervention with LSD adjustment and p adjusted for the following variables: marital status of the aged, relationship with the aged, time living with the aged and days as caregiver

The SHARE intervention did significantly affect the caregivers’ WHOQOL-OLD scores(Table 4). For example, at baseline, the autonomy scores were almost 17 points higher in the CG (*p*=.004) and, in the same group, they were remarkably reduced (p=.036) at baseline and at year 1, although they were markedly increased (p=.010) at year 1 in the IG. While the Social Participation scores did not remarkably differ between the CG and IG at baseline and at month 2, the IG scores were remarkably reduced(*p*<.001) between baseline and year 1. No statistically significant changes in the QoL scores were observed across the other four WHOQOL-OLD facets.

**Table 4:** Effect of SHARE on the family caregivers’ WHOQOL-OLD scores (n=15). PortoAlegre, RS, Brazil, 2018

Facet*	Item	CG^†^ (n=9)	IG^‡^ (n=6)	*p* ^§^
Sensory Abilities	Baseline^||^	71.8±7.0	74.8±7.5	0.821
	Month 2^||^	77.1±6.8	84.3±11.1	0.423
	Year 1^||^	75.9±7.6	85.8±7.6	0.348
	Month 2 - Baseline	5.22 (-6.01 to 16.5)	9.52 (-24.3 to 43.3)	
	*p* ^¶^	0.362	0.581	
	Year 1 - Month 2	-1.15 (-8.6 to 6.29)	1.52 (-20.5 to 23.6)	0.800
	*p* ^¶^	0.763	0.892	
	Year 1 - Baseline	4.08 (-4.27 to 12.4)	11.0 (-11.2 to 33.3)	
	*p* ^¶^	0.339	0.331	
Autonomy	Baseline^||^	66.1±6.0	49.2±2.2	0.004
	Month 2^||^	58.8±4.3	60.4±3.1	0.651
	Year 1^||^	55.9±4.7	58.5±3.9	0.659
	Month 2 - Baseline	-7.3 (-17.2 to 2.53)	11.1 (3.9 to 18.4)	
	*p* ^¶^	0.145	0.003	
	Year 1 - Month 2	-2.89 (-11.4 to 5.62)	-1.96 (-10.4 to 6.5)	0.004
	*p* ^¶^	0.506	0.648	
	Year 1 - Baseline	-10.2 (-19.7 to-0.68)	9.18 (2.22 to 16.1)	
	*p* ^¶^	0.036	0.010	
Past, Present and Future Activities	Baseline^||^	65.5±3.9	65.6±4.1	0.990
	Month 2^||^	66.8±3.3	71.5±2.6	0.210
	Year 1^||^	68.5±3.1	70.3±2.5	0.619
	Month 2 - Baseline	1.27 (-5.44 to 7.99)	5.89 (-4.71 to 16.5)	
	*p* ^¶^	0.710	0.276	
	Year 1 - Month 2	1.77 (-6.48 to 10.0)	-1.15 (-5.81 to 3.50)	0.714
	*p* ^¶^	0.675	0.626	
	Year 1 - Baseline	3.04 (-4.77 to 10.9)	4.73 (-6.23 to 15.7)	
	*p* ^¶^	0.446	0.398	
Social Participation	Baseline^||^	59.1±4.1	63.4±2.0	0.315
	Month 2^||^	59.0±4.4	62.7±1.9	0.383
	Year 1^||^	59.6±5.1	56.7±3.5	0.637
	Month 2 - Baseline	-0.03 (-10.3 to 10.2)	-0.68 (-6.35 to 4.99)	
	*p* ^¶^	0.996	0.814	
	Year 1 - Month 2	0.54 (-11.8 to 12.8)	-6.04 (-13.9 to 1.86)	0.529
	*p* ^¶^	0.931	0.134	
	Year 1 - Baseline	0.52 (-11.8 to 12.8)	-6.72 (-10.3 to -3.12)	
	*p* ^¶^	0.935	<0.001	
Death and Dying	Baseline^||^	58.0±8.9	69.9±7.3	0.368
	Month 2^||^	57.9±9.8	61.1±5.4	0.739
	Year 1^||^	54.0±8.5	63.8±6.2	0.330
	Month 2 - Baseline	-0.12 (-9.20 to 8.96)	-8.74 (-31.2 to 13.8)	
	*p* ^¶^	0.979	0.447	
	Year 1 - Month 2	-3.87 (-17.9 a 10.2)	2.63 (-7.90 to 13.2)	0.687
	*p* ^¶^	0.590	0.624	
	Year 1 - Baseline	-3.98 (-16.8 to 8.8)	-6.10 (-28.7 to 16.5)	
	*p* ^¶^	0.542	0.597	
Intimacy	Baseline^||^	59.6±7.1	65.9±1.4	0.319
	Month 2^||^	66.7±7.5	62.8±5.6	0.633
	Year 1^||^	60.6±6.3	61.6±5.6	0.906
	Month 2 - Baseline	7.15 (-9.24 to 22.5)	-3.8 (-13 to 6.85)	
	*p* ^¶^	0.363	0.543	
	Year 1 - Month 2	-6.13 (-20.1 to 7.84)	-1.27 (-15.3 to 12.7)	0.552
	*p* ^¶^	0.390	0.859	
	Year 1 - Baseline	1.02 (-13.3 to 15.4)	-4.35 (-15.6 to 6.95)	
	*p* ^¶^	0.889	0.450	
Overall	Baseline^||^	63.4±2.9	64.8±2.0	0.710
	Month 2^||^	64.4±2.7	67.2±1.8	0.207
	Year 1^||^	62.4±2.7	66.1±2.6	0.223
	Month 2 - Baseline	1.03 (-2.89 to 4.85)	2.34 (-4.04 to 8.72)	
	*p* ^¶^	0.598	0.472	
	Year 1 - Month 2	-1.95 (-6.15 to 2.24)	-1.04 (-5.27 to 13.19)	0.896
	*p* ^¶^	0.361	0.629	
	Year 1 - Baseline	-0.93 (-6.68 to 4.83)	1.29 (-6.09 to 8.69)	
	*p* ^¶^	0.752	0.731	

*Estimated means ± standard error; ^†^CG = Control Group; ^‡^IG = Intervention Group; ^§^Effect of the intervention between the groups by the General Estimated Equations (GEE) model with Least Significant Difference (LSD) adjustment. Adjusted p for the following variables: marital status of the aged, relationship with the aged, time living with the aged and days as caregiver; ^||^Baseline: 7 days after discharge; Month 2: two months after discharge; Year 1: one year after discharge; ^¶^Effect of the intragroup GEE intervention with LSD adjustment and p adjusted for the following variables: marital status of the aged, relationship with the aged, time living with the aged and days as caregiver.

## Discussion

This RCT study focuses on the effects of a tailored educational intervention among family caregivers of Brazilian aged stroke survivors. One year after the stroke survivors had been discharged from SCU-Stroke, statistically significant differences were observed in the family caregivers’ Quality of Life. Our most poignant finding was that the Social Relationships and Autonomy scores consistently favored caregivers who *did* receive the SHARE intervention.

In a German RCT^36^ conducted over a 6-month period, family caregivers of aged stroke survivors were offered 15 educational sessions about stroke and survivor rehabilitation, as well as how to circumvent their own mental distress and burden. Prior to discharge, all such strategies led to significant improvements in the caregivers’ physical (p<.01) and environmental (p<.01) QoL. Six months after discharge, the caregivers’ psychological(p<.05), social (p<.05) and environmental (p<.01) QoL further improved.

Others^37^ have reported significant improvements in the caregivers’ social functioning three months after discharge (p=0.02). A caregiver-oriented intervention program in this Taiwanese study consisted in offering health education, intensive discharge planning and three months of HVs to identify or solve problems, as well as telephone support. In Hong Kong^17^, a transitional care program offered 4 weeks of education about stroke, stroke survivor physical exercises, medications and diet, as well as caregiver resilience building and emotional management. There were also family meetings, HVs and telephone calls. Four weeks after discharge, the caregivers’ physical (p=.002) and mental (p=.005) QoL improved significantly. Contrary to SHARE, the program developed in Hong Kong^17^ addresses physical rehabilitation of the stroke survivors and the caregivers’ psychological needs and delivers a combination of care measures through a multidisciplinary team. These enhancements may explain the absence of physical QoL differences over time in our study.

In a training program in Portugal called InCARE, the caregivers that received guidance on care activities for three months after discharge through HVs and telephone calls reported borderline statistically significantly higher mental QoL levels (p=.050)^38^. In a cross-sectional study conducted with family caregivers of stroke patients in Luxembourg two years after the stroke, the overall scores in the psychological domain were lower in WHOQOL-BREF^39^. In our case, the scores in this domain were highest among our IG and second highest overall in our CG over time. Our findings may be related to filial responsibility, in which caring for one’s aging parents is a moral duty and a cultural expectation. Such expectations are prevalent in Brazilian, Asian and Latin societies^40^ ^-^ ^41^.

Although the SHARE intervention exerted significant positive effects on the Social Relationships in the IG *per se*, the Social Participation scores in the IG were significantly reduced between baseline and year 1. The focus of this domain is routine social activities in one’s own community. Perhaps, the family caregivers in this study most longed to maintain their external social relationships with, for example, friends. A study of aged people in southeastern Brazil^42^ revealed an association between QoL and self-esteem on both such QoL measures (p<.001). The Social Relationships domain had the highest mean scores(71.19±14.65), while the scores in the Social Participation facet were lower among older caregivers (63.06±16.68). As such, authors^42^ argue that it is important for health professionals and family members to encourage older caregivers to keep participating in community activities to nurture their social contacts.

Our findings reinforce the positive effect of providing emotional support for family caregivers related to maintaining their own personal activities, self-care and decision-making. During the HVs with the IG, the INs placed great emphasis on sharing caregiving responsibilities with other family members, paying attention to one’s own physical and mental health, and reserving time for oneself and for leisure activities.

Delivery of the SHARE intervention was associated with significant differences in Social Relationships and Autonomy, which favored the IG. In Brazil, transitional care programs need the participation of health professionals, aged people and family caregivers to carry out discharge and care planning for a successful hospital-home transition^43^. Hence, nurse-led guidelines around caregiving activities and follow-up with family caregivers are essential^17^ ^,^ ^44^.

Cohort^45^ and prospective^46^ studies outside Brazil and a cross-sectional study conducted in Brazil^47^ indicate that the caregivers’ QoL is often lower in the Environmental domain. We found this to be the case among the CG and IG caregivers. Physical security, financial resources, access to information, and transportation are vital aspects of everyday life^30^. The SHARE intervention would not have been sufficient to help our IG overcome the unfavorable socioeconomic conditions they face every day. These include financial difficulties, unemployment, violence and lack of access to good quality health care services and formal support networks. Researchers in other developing countries suggest that low income, health problems, low schooling levels and being a caregiver are predictors of poorer QoL^45^ ^,^ ^48^. All such characteristics were prevalent in the IG and CG in this study. The caregivers’ sense of resilience would be an important consideration in a future SHARE study. Resilience would speak to the family caregivers’ ability to positively adapt to their new roles despite circumstantial adversities^49^.

The Brazilian Home Care Policy currently recommends an initial HV within seven to 30 days for patients requiring higher levels of care needs due to, for example, having experienced a stroke^50^. The SHARE intervention involved HVs within this prescribed time period and led to remarkable improvements in the caregivers’ QoL. Early caregiver support is thus essential.

Another proven important aspect of the SHARE intervention lies in the instability of the CG scores in nearly all the WHOQOL-BREF domains. The scores in the CG were higher at baseline and dropped over time, generally presenting higher variability. The scores in the IG, while lower at baseline, were more stable over time. This may have been partly due to the support and guidance provided by SHARE nurses who presumably had a better anticipatory understanding of what caregiving entails for aged stroke survivors. All caregivers in this study were providing support to first-time stroke survivors.

This RCT has some limitations. First and foremost, the caregivers were recruited from a single Brazilian region with unique social and economic circumstances. It is unfortunate that we had no socioeconomic data pertaining to household characteristics. The caregivers were also working with health professionals with highly specialized knowledge about stroke survivor care. Only answering “what works” without empirical attention to household characteristics does not shed light on the everyday caregiving context. Our findings cannot be generalized beyond the caregivers included in this study. The quantitative research questions draw the attention to a specific population segment (family caregivers) and to a specific living environment (own home) but cannot aptly speak to diversity in the caregivers’ everyday living circumstances^51^.

In a future study, more inclusive sampling among survivors discharged from non-specialized institutions across multiple geographic regions is warranted. We also most certainly need to interview caregivers about their everyday socioeconomic and environmental constraints.

Doing so is likely to shed greater light on our Overall QoL findings, with these favoring the IG in our comparisons between month 2 and year 1 scores. When conducting RCTs, using mixed methods is a means to expand what can be learned from an intervention research study. The participants’ voices need to be heard and their shared experiences need to be drawn upon to better understand effectiveness of the intervention. It is necessary to go beyond answering whether an intervention works, to answering how and under what circumstances the results are achieved^52^. Quality of Life is a person’s perception of their position in life^31^.

It is also worth noting that the way in which QoL is measured in published intervention studies varies considerably across countries. We lacked points of comparison for changes in the WHOQOL-BREF scores over time. Our somewhat pallid internal consistency coefficients for its Psychological and Social Relationships domains are cases-in-point. Nonetheless, it is our position that what we have learned about the power of educational support to effect positive changes in caregivers’ QoL deficits far outweighs these shortcomings. We hope that the findings of this study spurs researchers on to adopt WHOQOL-BREF in future intervention studies so that all such comparisons can be readily made.

## Conclusion

The SHARE intervention exerted a statistically significant effect on family caregivers’ QoL with respect to their social relationships and autonomy. Interventions to support physical provision of care and QoL are important. Gains in knowledge about stroke survivor care and care delivery alone are not sufficient. The caregivers’ knowledge and QoL should be assessed before aged stroke survivors are discharged. In Brazil, there are no formal long-term support service programs for safeguarding caregivers’ QoL. Caregivers need to return home to adequate support systems so that they have time to care for themselves. Multidisciplinary teams that can work with caregivers in their own homes are necessary. Ideally, such teams would include a broad network of healthcare professionals, family members and friends. Public policies that emphasize the importance of all such support programs are critical.
